# Down-Regulation of miR-7 in Gastric Cancer Is Associated With Elevated LDH-A Expression and Chemoresistance to Cisplatin

**DOI:** 10.3389/fcell.2020.555937

**Published:** 2020-09-22

**Authors:** Hui-Fang Jin, Ju-Feng Wang, Ming Shao, Kailu Zhou, Xiao Ma, Xian-Ping Lv

**Affiliations:** ^1^Department of Bloood Transfusion, The First Affiliated Hospital of Zhengzhou University, Zhengzhou, China; ^2^Department of Oncology, Henan Cancer Hospital, The Affiliated Cancer Hospital of Zhengzhou University, Zhengzhou, China; ^3^Medical College of Zhengzhou University, Zhengzhou, China; ^4^Department of Ultrasound, The First Affiliated Hospital of Zhengzhou University, Zhengzhou, China

**Keywords:** gastric cancer, MiR-7, LDH-A, glycolysis, chemosensitivity

## Abstract

MicroRNAs (miRNAs) are dysregulated in the context of many cancer types, making them potentially ideal diagnostic or therapeutic targets in patients in which they are aberrantly expressed. In the present study, we found miR-7 to be downregulated in gastric cancer (GC), and we further determined its expression to be closely linked to GC sensitivity to the chemotherapeutic compound cisplatin. This effect appears to be at least partially attributable to the regulation of LDH-A, which is a miR-7 target gene and expression of LDH-A is negatively correlated with miR-7 expression in primary GC tumor samples. When upregulated, we also determined that miR-7 was able to inhibit the proliferation, colony formation, and glycolysis of GC cells owing to its regulation of LDH-A. Moreover, overexpression of miR-7 render cells more sensitive to cisplatin. Our results thus provide novel evidence that miR-7 is a key mediator of GC growth and chemosensitivity through its regulation of LDH-A, thus potentially highlighting this pathway as a therapeutic target for treating affected patients.

## Introduction

Gastric cancer (GC) is one of the deadliest forms of cancer, accounting for almost 10% of cancer-associated mortality globally (Ferlay et al., 2010). This high mortality is in part attributable to the fact that most patients are not diagnosed until they are in a more advanced stage of the disease (An et al., 2015). The primary therapeutic strategy for treating GC is chemotherapy (especially cisplatin treatment), however, drug resistance to cisplatin remains a major obstacle to therapeutic success in these patients. Therefore, the development of novel therapies for GC is essential in order to improve patient outcomes.

MicroRNAs (miRNAs) are short RNA molecules that lack coding capacity but that are able to regulate key biological activities related to tumor development and progression, in addition to normal physiological processes (Krutzfeldt et al., 2006; Cho, 2007; Zheng et al., 2010). The miR-7 miRNA, for example, has been shown to act as a tumor suppressor such that when expression of this miRNA is lost there is an increase in tumor progression, as has been reported in many different cancers (Zhao et al., 2013; Luo et al., 2015; Shi et al., 2015; Cao et al., 2016; Giles et al., 2016; Hua et al., 2016). miR-7 has been found to have a variety of potential target genes in this context, including IGFIR, FAK, RelA, CKS2, PAX6, and REGγ (Zhao et al., 2013; Luo et al., 2015; Shi et al., 2015; Cao et al., 2016; Giles et al., 2016; Hua et al., 2016), all of which are directly relevant to cancer. While it has been studied in the context of various cancers to date, the importance of miR-7 in GC remains to be studied.

Lactate dehydrogenase A (LDH-A) is an enzyme important for controlling glycolytic metabolism, and its expression is elevated in a range of human cancer types in a manner associated with worse patient outcomes. Glycolysis relies upon the breakdown of glucose molecules into pyruvate, producing a total of two ATP molecules per glucose (Vander Heiden et al., 2009). As tumor cells rapidly utilize glucose, they additionally produce large quantities of pyruvate even when oxygen is available. LDH-A is then able to metabolize these pyruvate stores into lactate (Fantin et al., 2006; Le et al., 2010; Allison et al., 2014). As such, inhibition of LDH-A has been shown to induce cancer cell death and it appears to be required for survival of a range of cancer cell types (Fantin et al., 2006; Le et al., 2010; Allison et al., 2014). All other points raised below have now been mostly addressed – however, there are a few significant points indicated above that need addressing.

Herein, we investigated the role of miR-7 in human GC, revealing it to be downregulated in tumor samples. We then further assessed the molecular mechanisms whereby miR-7 influences GC through a series of experiments in which we assessed the effects of miR-7 expression levels on tumor cell proliferation, metabolism, and drug resistance. Through these experiments, we hope to yield novel insights into the metabolic and molecular basis of GC, therefore to facilitate the development of future therapeutic treatment strategies.

## Materials and Methods

### Tissue Samples

A total of 32 GC samples were isolated following their surgical resection from patients treated at the First Affiliated Hospital of Zhengzhou University, Zhengzhou, China. A pathologist confirmed that all samples were tumors or normal tissue as appropriate, and samples were snap frozen prior to eventual analysis. The Research Ethics Committee of Zhengzhou University approved this study, with all patients giving written informed consent.

### Cell Culture

Human gastric cancer cell lines SGC7901 and BGC823 were purchased from the Type Culture Collection of the Chinese Academy of Sciences. Cells were cultured in Dulbecco’s modified Eagle medium (DMEM) supplemented with 10% fetal bovine serum (FBS). Stable cell lines were established by infecting lentivirus into cells and selected by puromycin. To stably overexpress miR-7 in GC cells, the lentiviral packaging kit was used (Thermo Fisher Scientific). Lentivirus carrying miR-7 or negative control (miR-NC) was packaged following the manufacturer’s manual.

### qRT-PCR

Trizol (Takara) was used to extract sample RNA based on provided directions (Li et al., 2019; Jin et al., 2020) LDH-A To quantify the mRNA levels of LDH-A, RNAs were transcribed using oligodT primer using RT Reagent Kit (Vazyme, China). To measure miR-7 expression levels, RNAs were transcribed by stem-loop RT primer using RT Reagent Kit (Vazyme, China). qRT-PCR was performed using SYBR Green Master Mix (Vazyme, China) on a 7500HT system (Applied Biosystems, United States). β-actin or U6 levels were used as an internal control, respectively. miR-7 and U6 primer kit were purchased from Riobio (Guangzhou, China). Primers for genes as below:

LDH-A: Forward (GGATCTCCAACATGGCAGCCTT), Reverse (AGACGGCTTTCTCCCTCTTGCT); and β-actin: Forward (CACCATTGGCAATGAGCGGTTC), Reverse (AGGTCTTTGCGGATGTCCACGT).

### Cell Proliferation Assay

To determine the effects of miR-7 on growth of gastric cancer cells, cells (2,500 cells per well) were seeded in 96-well plates and incubated in corresponding medium supplemented with 10% FBS. The absorptions of the cells (Absorbance value at 450 nm) were measured using a CCK8 kit (Dojindo Laboratories, Japan) according to the manufacturer’s instruction at different indicated time points.

### Colony Formation Assay

A total of 300 cells were added to wells of 12 well plates, with media changed every third day. After 15 days, cells underwent methanol fixation and staining using 0.1% crystal violet (Sigma-Aldrich). Colonies were then counted for three independent experiments.

### Assessment of Cellular Metabolism

Cells were seeded into 96-well plates with 200 μL media each well. To determine the levels of glucose and lactate in BGC823 and SGC7901 cells, the supernatants of cell culture media(miR-7 or miR-NC) were collected and assayed for glucose and lactate levels by using glucose assay kit and lactate assay kit (BioVision, San Francisco, United States) according to the manufacturer’s instructions. The values at different time periods were analyzed by the OD values. Glucose consumption and lactate production were calculated based on the standard curve, and normalized to the cell number.

### Immunoblotting

RIPA buffer containing protease inhibitors was used to lyse cells, after which a BCA assay (Beyotime Institute of Biotechnology, Jiangsu, China) was used for protein quantification. Protein was then separated using SDS-PAGE, transferred to a PVDF membrane, and subjected to immunoblotting with appropriate reagents. Protein signal was visualized with an ECL Detection System (Thermo Scientific, IL, United States). Antibody against LDH-A was from Abcam (Abcam, United States, catalog mumber: ab101562), and antibody against β-actin was from Santa Cruz Biotechnology (Santa Cruz, United States, catalog mumber: sc-69879), respectively.

### Luciferase Reporter Assay

The LDH-A 3′UTR sequence was generated and added to the pMIR-reporter luciferase vector (Ambion), with a mutant construct also being created in which the normal miR-7 binding site (UCUUCC) was changed to UGAUGA. Then wild type and mutant constructs were validated by DNA sequencing, and cells were seeded in 24-well plates and co-transfected with 0.3 μg of luciferase reporter plasmids, 0.1 μg of Renilla luciferase reporter (internal control) and equal amounts (50 nM) of miR-7 or miR-NC using Lipofectamine 3000 (Invitrogen). Firefly and Renilla luciferase activities were measured 24 h after transfection using a dual luciferase assay kit (Promega).

### *In vitro* Chemosensitivity

A total of 4000 cells were added to wells of 96-well plates overnight, after which 1.25 – 80 μM of freshly made cisplatin (Sigma-Aldrich) was used to treat cells for 48 h, followed by CCK-8 analysis as above.

### Apoptosis Assessment

Cells were stained using PE-Annexin V and propidium iodide (BD Pharmingen), after which they were assessed on a flow cytometer (FACS Canto II, BD Biosciences), with FlowJo used for data analysis.

### Measurement of Caspase-3 Activity

A Beyotime caspase-3 activity kit was used based on provided directions, assessing caspase-3 activity using cell lysates added to 96-well plates with a buffer containing the appropriate caspase-3 substrate (Ac-DEVD-pNA). Assays were performed on 96-well plates by incubating 10 μL protein of cell lysate per sample (10 μg) in 80 μL reaction buffer containing 10 μL caspase-3 substrate (Ac-DEVD-pNA; 2 mM) at 37°C for 2 h. The reaction was then measured at 405 nm for absorbance.

### *In vivo* Models

Nude BALB/c mice (4-6-weeks-old) from the Shanghai Laboratory Animal Center were housed for 1 week prior to experimental use, after which they were subcutaneously implanted into both flanks with 5 × 10^6^ cells BGC823 cells stably expressing miR-7 or miR-NC (Negative Control) in 100 μl. Calipers were used to measure tumors every other day, and tumor volume was calculated as 0.5 × Length × Width^2^. After 24 days, animals were euthanized and tumors were excised for western blotting and qRT-PCR. All animal studies were consistent with the Guide for the Care and Use of Laboratory Animals.

### Statistical Analysis

GraphPad Prism 5 (CA, United States) was used for statistical analysis of triplicate experiments. Pearson’s correlation test was used to gage the relationship between miR-7 and LDH-A expression in GC, with *t*-tests used for other statistical comparisons. *P* < 0.05 was the significance threshold.

## Results

### Human GC Tissues Exhibit Reduced miR-7 Expression

**FIGURE 1:**
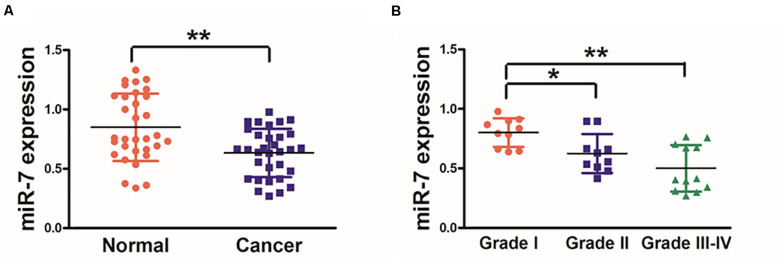
Human GC tissues exhibit decreased miR-7 expression. **(A)** The expression of miR-7 was examined via qRT-PCR in 32 paired GC and adjacent normal stomach tissue samples, with U6 used for normalization. **(B)** A pathologist conducted histological examination of all samples, and relative miR-7 expression as a function of tumor stage is shown. Data are means ± SD. *P < 0.05; **P < 0.01.

We first explored the relevance of miR-7 in GC tissues by assessing the expression of this miRNA via qRT-PCR in 32 paired GC tumor and adjacent normal tissues, revealing significantly lower miR-7 levels in tumor samples (Figure 1A). These samples were also subjected to staging by a clinical pathologist, revealing that WHO stage III-IV GC tissue samples exhibited significantly lower miR-7 expression than stage I-II samples, suggesting more pronounced miR-7 downregulation at later stages of disease (Figure 1B). In addition, miR-7 levels were markedly lower in the patients with lymph node metastasis than those in the patients without lymph node metastasis (Table 1). These findings thus indicate that miR-7 downregulation occurs within GC tissues.

**TABLE 1:** Comparison of clinicopatothologic factors and normalized expression of miR-7 in 32 pairs of GC.

**Gastric cancer**	**Number**	Normalized expression of miR-7*
**Age (year)**		
<60	20	0.6748 (0.2708–0.8991)
≥60	12	0.6624 (0.3112–0.9790)
*P*-value		0.5356
**Sex**		
Male	19	0.6677 (0.2708–0.9790)
Female	13	0.5862 (0.2990–0.8982)
*P*-value		0.3127
**Lymph node**		
Negative	15	0.7841 (0.6315–0.9790)
Positive	17	0.4808 (0.2708–0.8367)
*P*-value		<0.01
**TNM stage**		
Grade I	10	0.7941 (0.6393–0.9790)
Grade II	10	0.5581 (0.4164–0.8982)
Grade III–IV	12	0.4161 (0.2708–0.7667)
*P*-value		<0.05

*Median of normalized expression of miR-7 with 25th-75th precentile in parenthesis.

### MiR-7 Suppresses GC Cell Proliferation and Glycolysis

**FIGURE 2:**
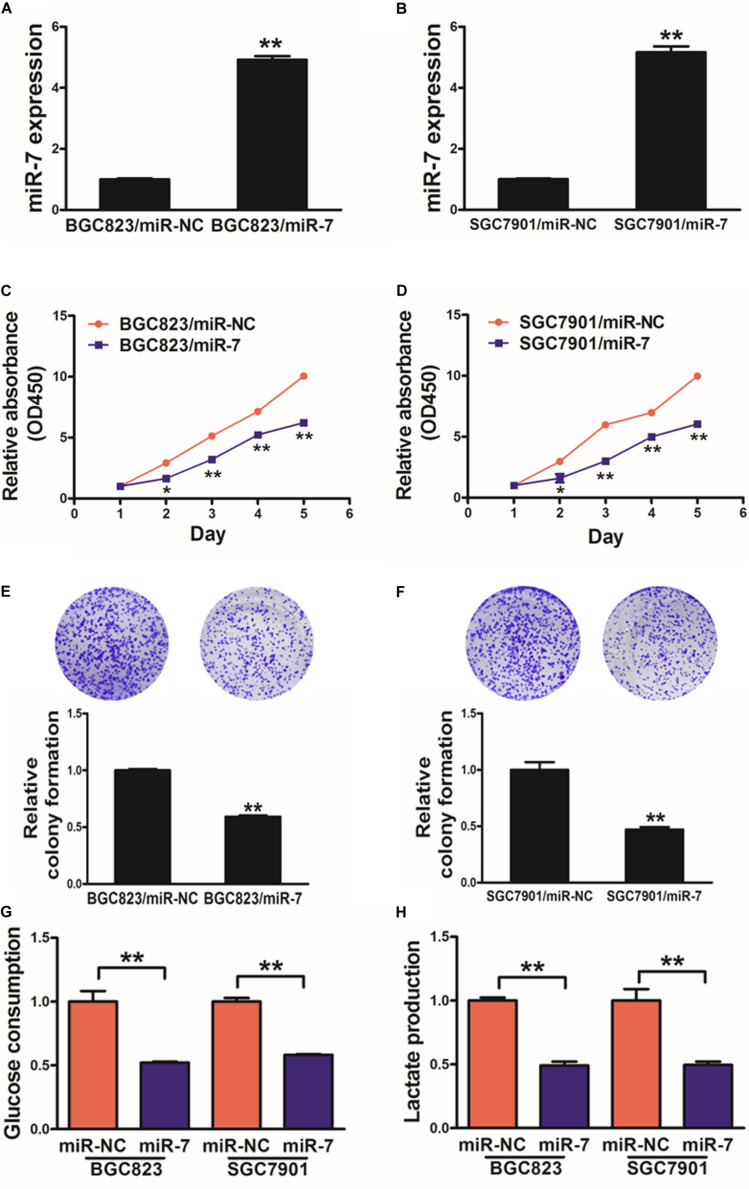
miR-7 expression disrupts GC cell proliferation and glycolysis. **(A,B)** Relative miR-7 levels were assessed via qRT-PCR in BGC823 or SGC7901 cells transduced to stably express miR-7 or miR-NC. **(C,D)** A total of 2000 cells were added to wells of 96-well plates, and a CCK-8 kit was used to assess proliferation daily. **(E,F)** A colony formation assay was conducted using stably transduced cells. **(G,H)** Glucose utilization and lactate production were assessed for all cells. Three independent experiments were conducted in triplicate. Data are means ± SD. *P < 0.05; **P < 0.01.

We next assessed the mechanistic role of miR-7 in GC using the BGC823 and SGC7901 cell lines, which we induced to stably express either miR-7 or negative control (NC) via lentiviral transduction. qRT-PCR was then used to confirm elevated miR-7 expression in the BGC823 and SGC7901 cells transduced with the miR-7 lentivirus, confirming successful model generation (Figures 2A,B).

When these cells were analyzed in detail, we determined that miR-7 overexpression decreased proliferation and colony formation in relative to NC cells (Figures 2C–F, Supplementary Figure S1). We additionally assessed the metabolism of these cells in the context of miR-7 overexpression, revealing it to impair the utilization of glucose and production of lactate in both cell lines (Figures 2G,H). Moreover, inhibition of miR-7 in BGC823 induced cell proliferation, colony formation activity and lactate production (Supplementary Figure S2). These results thus indicate that miR-7 suppresses GC cell proliferation and glycolysis, thus acting as a tumor suppressor.

### miR-7 Inhibits LDH-A Expression in GC Cells

**FIGURE 3:**
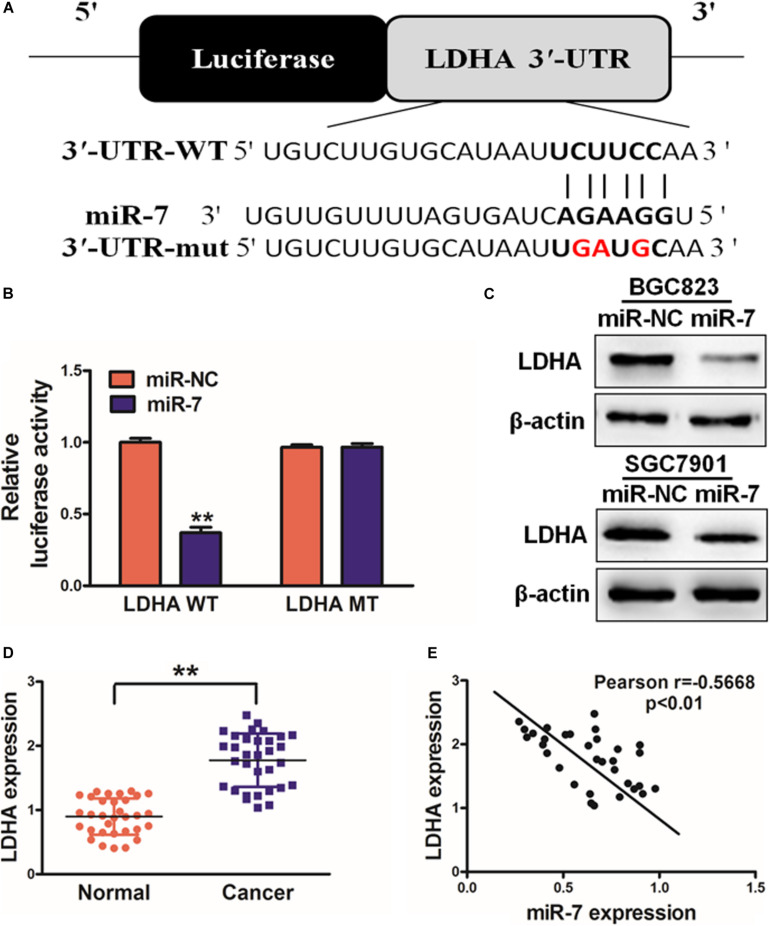
miR-7 targets LDH-A in GC tissues. **(A)** Human miR-7 sequence aligned to the putative 3′UTR binding site in human *LDH-A*, with the mutated *LDH-A* sequence also shown (mutated nucleotides are red). **(B)** BGC823 were transfected with WT or mutant (MT) LDH-A 3′-UTR luciferase reporters along with miR-7 or miR-NC, and then after 24 h luciferase activity was assessed, revealing a miR-7-dependent suppression of WT promoter activity. Three independent experiments were conducted in triplicate. **(C)** Western blotting revealed reduced LDH-A expression in cells overexpressing miR-7. **(D)** LDH-A expression in human control and GC samples, measured via qRT-PCR with β-actin used for normalization. **(E)** The correlation between LDH-A and miR-7 in GC patient tissues was assessed via Pearson correlation analysis. Data are means ± SD. **P < 0.01.

In order to better understand how miR-7 influences GC growth and metabolism, we next used TargetScan^1^ to predict possible miR-7 target genes, identifying LDH-A as one such target (Figure 3A). To test this possibility, we next co-transfected BGC823 cells using miR-7 or NC along with a luciferase reporter construct containing the WT or mutant (MT) miR-7 binding sequence in the LDH-A 3′UTR. After 24 h, luciferase activity was assessed, revealing that miR-7 signfiicantly reduced WT but not MT luciferase signal, confirming binding activity (Figure 3B). LDH-A protein levels were also assessed by western blotting, confirming decreased levels of this protein in cells overexpressing miR-7 (Figure 3C). This thus suggested that miR-7 can directly target LDH-A in GC cells. This was further confirmed when we observed elevated LDH-A expression in GC tissue samples relative to normal controls (Figure 3D). Pearson’s correlation analysis further confirmed that LDH-A and miR-7 expression were inversely correlated in GC tissue samples, further supporting this result (Figure 3E; *r* = −0.5668, *p* < 0.01).

### miR-7 Overexpression Inhibits GC Cell Proliferation and Glycolysis via Targeting LDH-A

**FIGURE 4:**
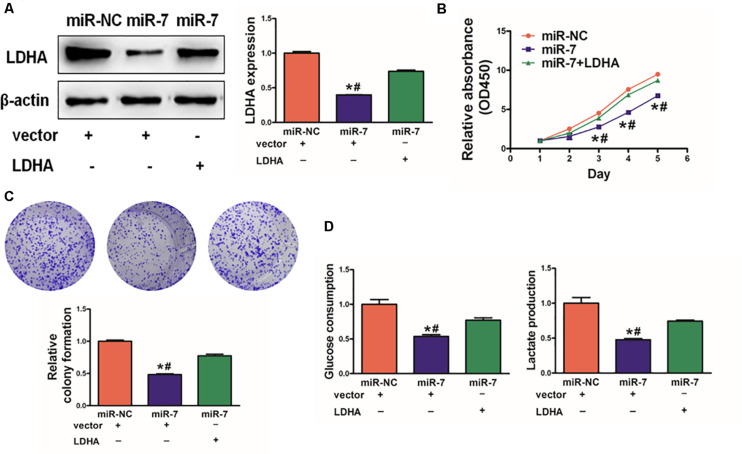
LDH-A overexpression overcomes the inhibition effects of miR-7. **(A)** LDH-A expression was increased via plasmid transient transfection, 72 h later, protein were extracted, and results as shown. **(B)** When miR-7 was overexpressed, cell proliferation decreased, whereas co-overexpression of LDH-A rescued this phenotype. **(C)** A colony formation assay was performed with the indicated cells. **(D)** Glucose utilization and lactate production were assessed for all indicated cells. Data are means ± SD. *P < 0.05 vs control; *^#^P* < 0.05 vs miR-7 and LDH-A overexpression. **P < 0.01.

As LDH-A is known to play a key role in tumor growth, knockdown with LDHA siRNA decreased cell proliferation, colony formation activity, and lactate production activity (Supplementary Figure S3). We next assessed its role in the context of miR-7 expression in BGC823 cells. To investigate whether LDHA, the target gene of miR-7, was involved in miR-7-regulated cell proliferation and colony formation, we overexpressed LDHA protein levels in BGC823/miR-7 cells by infecting cells with LDHA overexpression plasmids (vector control: pcDNA3-empty plasmid, 1μg), and restoration of LDHA rescued by WB (Figure 4A). We found that when LDH-A expression was induced in cells overexpressing miR-7, this rescued the miR-7-induced inhibition in cell proliferation (Figure 4B).

Consistent with this, we found that miR-7 significantly impaired colony formation and glycolysis in BGC823 cells, whereas inducing LDH-A expression partially reversed these phenotypes (Figures 4C,D). Together, these findings thus indicated that miR-7 overexpression can suppress the growth and glycolysis of GC cells at least in part via LDH-A inhibition.

### miR-7 Overexpression Elevated GC Cell Cisplatin Chemosensitivity via Targeting LDH-A

**FIGURE 5:**
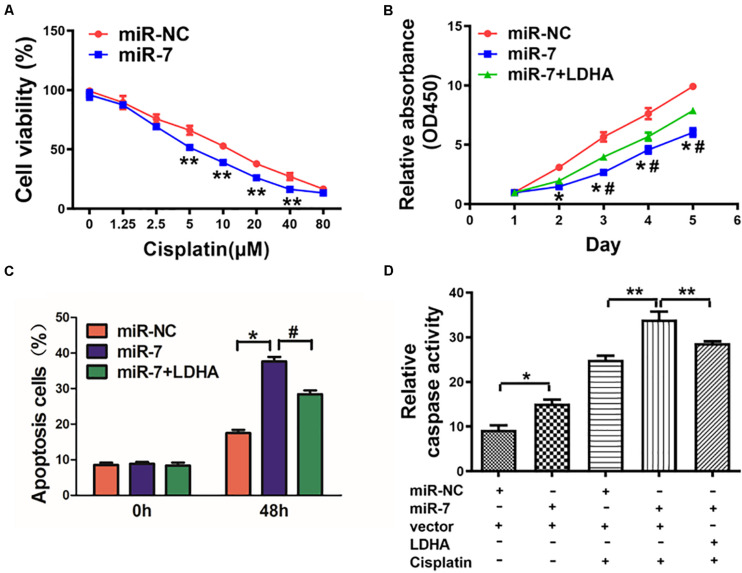
miR-7 overexpression increased GC cell cisplatin sensitivity via LDH-A inhibition. **(A)** BGC823 cells that had undergone stable transduction to express miR-7 or miR-NC were treated with the indicated amounts of cisplatin prior to CCK-8 assessment (48 h after cisplatin treatment). **(B)** BGC823 cells that had undergone stable transduction to express miR-7, miR-NC, or both miR-7 and LDH-A were treated with the indicated amounts of cisplatin (5 μM) prior to CCK-8 assessment. **(C,D)** Apoptosis was measured via flow cytometry in cells treated with or without cisplatin (5 μM). Three independent experiments were conducted in triplicate. Data are means ± SD. *P < 0.05 vs control; *^#^P* < 0.05 vs miR-7 and LDH-A overexpression. **P < 0.01.

Cisplatin resistance is a major driver of chemotherapy failure during GC treatment. We found that increasing miR-7 expression increased the chemosensitivity of BGC823 cells to cisplatin (Figure 5A). When these cells were induced to also express LDH-A and were then treated with 5 μM cisplatin, we found that enforced expression of LDH-A was sufficient to reverse this miR-7-dependent rise in cisplatin sensitivity (Figure 5B). We further analyzed the apoptotic death of these cells via flow cytometry and measuring the activity of caspase 3, revealing that miR-7 overexpression increased apoptosis and caspase-3 activation, whereas inducing LDH-A expression reversed this phenotype (Figures 5C,D). Together these findings thus demonstrate that miR-7 increases GC sensitivity to cisplatin-induced cell death.

### MiR-7 Impairs *in vivo* Tumor Growth

**FIGURE 6:**
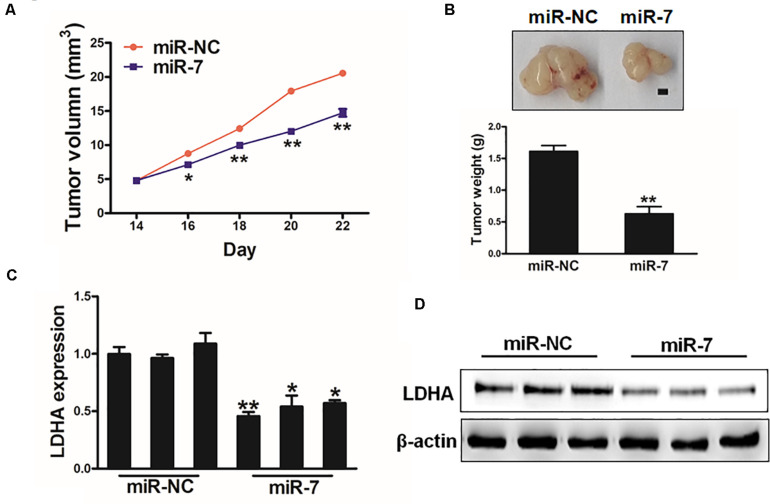
MiR-7 impairs *in vivo* tumor growth. **(A,B)** A total of 5 × 10^6^ BGC823 cells that had been stably transduced to express miR-7 or miR-NC were implanted subcutaneously in nude BALB/c null mice, after which tumor volumes were assessed over time. After 24 days, tumors were collected and weighed, revealing a lower weight for miR-7 overexpressing tumors. Scale bar = 1 mm. **(C,D)** LDH-A expression was decreased in miR-7 overexpressing tumors relative to NC controls as assessed via qRT-PCR. *P < 0.05; **P < 0.01.

We next explored the clinical relevant of miR-7 in GC in vivo using a murine xenograft model in which mice were implanted subcutaneously on both flanks with BGC823 cells stably expressing miR-7 or NC. Tumor sizes were measured regularly, revealing significantly larger NC tumors relative to miR-7 tumors from weeks 2–4 of tumor growth (Figure 6A). Indeed, tumors in which miR-7 was stably expressed were significantly smaller than control tumors, with a smaller final tumor weight (Figure 6B). Consistent with our previous findings, LDH-A expression was significantly lower in tumors overexpressing miR-7 relative to NC tumors as measured by western blotting and qRT-PCR (Figures 6C,D). These find thus indicate that miR-7 can inhibit the growth of tumors and target LDH-A *in vivo*.

## Discussion

miRNAs have the ability to post-transcriptionally regulate a wide array of genes, with nearly a third of genes thought to undergo some form of miRNA-mediated regulation (Lu et al., 2005). Depending on the specific miRNA and context, these noncoding RNAs can serve to promote or suppress tumor growth, leading to their dysregulation in many cancers (Ling et al., 2013; Niu et al., 2017; Ge et al., 2018). miR-7 has been found in recent years to serve as a tumor suppressor in several tumor types, but its role in GC has not previously been demonstrated. Recent reports showed that miR-7 plays a certain role in gastric cancer and can be regulated by Circular RNA, long non-coding RNA or DNA methylation (Pan et al., 2018; Yang et al., 2018; Xin et al., 2020). Herein, we found miR-7 to be expressed at lower levels in GC tumors, and when overexpressed miR-7 inhibited the proliferation and glycolytic metabolism of GC cells, in addition to increasing their cisplatin sensitivity.

Aerobic glycolysis rates are often substantially increased in tumors, in what has been termed the Warburg effect, which is a major cancer hallmark (Cai et al., 2010; Sheng et al., 2012; Cui et al., 2014; Girgis et al., 2014; Yang et al., 2014; Wang et al., 2015). This shift in normal cellular metabolism can offer cancerous cells an advantage by allowing them to more readily utilize available energy to fuel their unrestrained growth. LDH-A is an essential enzyme in the glycolytic pathway, metabolizing pyruvate into lactate to assist with tumor growth. Indeed, LDH-A expression is elevated in a wide range of tumor types, and is correlated with worse patient outcomes (Cai et al., 2010; Sheng et al., 2012; Cui et al., 2014; Girgis et al., 2014; Yang et al., 2014; Wang et al., 2015). reported that colorectal cancer express higher levels of LDHA compared with adjacent normal tissue. Knockdown of LDHA resulted in decreased lactate and ATP production, and glucose uptake. Furthermore, Wang et al. found that miR-34a, miR-34c, miR-369-3p, miR-374a, and miR-4524a/b target LDHA and regulate glycolysis in cancer cells (Wang et al., 2015). In breast cancer cells, higher LDH-A expression can increase tumor cell growth and metastasis (Xiao et al., 2016). miRNAs have recently been shown to regulate LDH-A. Consistent with this, we found miR-7 to target LDH-A directly, with GC tissues exhibiting a corresponding negative correlation between LDH-A and miR-7 expression. Our results thus offer novel insight into a mechanism whereby miR-7 can suppress GC cancer progression via inhibiting LDH-A expression.

Although research into GC progression and development has offered unparalleled insight into the mechanisms of this deadly disease, at present its clinical treatment remains challenging owing in part to chemoresistance. miRNAs have recently been identified as potential regulators of chemosensitivity in many cancers. Fore example, miR-143 can increase the sensitivity of glioma cells to apoptotic death upon temozolomide treatment owing to its targeting of N-Ras, and miR-124 can similarly enhance the chemosensitivity of glioma cells via its regulation of both N-Ras and R-Ras (Shi et al., 2014; Wang et al., 2014). Consistent with their relevance to chemoresistance, we found that miR-7 overexpression rendered GC cells more sensitive to cisplatin-induced apoptosis in BGC823 cells with miR-7 to cisplatin was induced by apoptosis. As such, therapeutic interventions that increase miR-7 expression have the potential to help overcome cisplatin resistance in GC.

In conclusion, our results offer novel insight into the importance of miR-7 in GC, wherein it regulates LDH-A expression to control tumor progression. We found that miR-7 was able to constrain proliferation, glycolysis, and chemoresistance in GC cells at least in part owing to its ability to directly target LDH-A. With this improved understanding of the molecular mechanisms of GC, it may be possible to leverage this novel biomarker to improve GC patient diagnosis and therapeutic treatment in the future.

## Data Availability Statement

The original contributions presented in the study are included in the article/Supplementary Material, further inquiries can be directed to the corresponding author/s.

## Ethics Statement

The studies involving human participants were reviewed and approved by the Ethics Committee of Zhengzhou University. The patients/participants provided their written informed consent to participate in this study. The animal study was reviewed and approved by the Ethics Committee of Zhengzhou University.

## Author Contributions

H-FJ designed and performed the experiments and wrote the manuscript. J-FW, MS, KZ, and XM performed the qRT-PCR assay, immunoblotting analysis, and analyzed chemosensitivity assay. X-PL supervised the project. All authors contributed to the article and approved the submitted version.

## Conflict of Interest

The authors declare that the research was conducted in the absence of any commercial or financial relationships that could be construed as a potential conflict of interest.

## Footnotes

1 www.targetscan.org/vert_72/

## References

[B1] Allison S. J. Knight J. R. Granchi C. Rani R. Minutolo F. Milner J. (2014). Identification of LDH-A as a therapeutic target for cancer cell killing via (i) p53/NAD(H)-dependent and (ii) p53-independent pathways. *Oncogenesis* 3:e102. 10.1038/oncsis.2014.16 24819061 PMC4035693

[B2] An Y. Zhang Z. Shang Y. Jiang X. Dong J. Yu P. (2015). miR-23b-3p regulates the chemoresistance of gastric cancer cells by targeting ATG12 and HMGB2. *Cell Death Dis.* 6:e1766. 10.1038/cddis.2015.123 25996293 PMC4669702

[B3] Cai Z. Zhao J. S. Li J. J. Peng D. N. Wang X. Y. Chen T. L. (2010). A combined proteomics and metabolomics profiling of gastric cardia cancer reveals characteristic dysregulations in glucose metabolism. *Mol. Cell. Proteom.* 9 2617–2628. 10.1074/mcp.m110.000661 20699381 PMC3101851

[B4] Cao Q. Mao Z. D. Shi Y. J. Chen Y. Sun Y. Zhang Q. (2016). MicroRNA-7 inhibits cell proliferation, migration and invasion in human non-small cell lung cancer cells by targeting FAK through ERK/MAPK signaling pathway. *Oncotarget* 7 77468–77481. 10.18632/oncotarget.12684 27764812 PMC5363598

[B5] Cho W. C. (2007). OncomiRs: the discovery and progress of microRNAs in cancers. *Mol. Cancer* 6:60. 10.1186/1476-4598-6-60 17894887 PMC2098778

[B6] Cui J. Shi M. Xie D. Wei D. Jia Z. Zheng S. (2014). FOXM1 promotes the warburg effect and pancreatic cancer progression via transactivation of LDHA expression. *Clin. Cancer Res.* 20 2595–2606. 10.1158/1078-0432.ccr-13-2407 24634381 PMC4024335

[B7] Fantin V. R. St-Pierre J. Leder P. (2006). Attenuation of LDH-A expression uncovers a link between glycolysis, mitochondrial physiology, and tumor maintenance. *Cancer Cell* 9 425–434. 10.1016/j.ccr.2006.04.023 16766262

[B8] Ferlay J. Shin H. R. Bray F. Forman D. Mathers C. Parkin D. M. (2010). Estimates of worldwide burden of cancer in 2008: GLOBOCAN 2008. *Int. J. Cancer* 127 2893–2917. 10.1002/ijc.25516 21351269

[B9] Ge X. Pan M. H. Wang L. Li W. Jiang C. He J. (2018). Hypoxia-mediated mitochondria apoptosis inhibition induces temozolomide treatment resistance through miR-26a/Bad/Bax axis. *Cell Death Dis.* 9:1128. 10.1038/s41419-018-1176-7 PMC6233226 30425242

[B10] Giles K. M. Brown R. A. Ganda C. Podgorny M. J. Candy P. A. Wintle L. C. (2016). microRNA-7-5p inhibits melanoma cell proliferation and metastasis by suppressing RelA/NF-kappaB. *Oncotarget* 7 31663–31680. 10.18632/oncotarget.9421 27203220 PMC5077967

[B11] Girgis H. Masui O. White N. M. Scorilas A. Rotondo F. Seivwright A. (2014). Lactate dehydrogenase A is a potential prognostic marker in clear cell renal cell carcinoma. *Mol. Cancer* 13:101. 10.1186/1476-4598-13-101 24885701 PMC4022787

[B12] Hua K. Jin J. Zhang H. Zhao B. Wu C. Xu H. (2016). MicroRNA-7 inhibits proliferation, migration and invasion of thyroid papillary cancer cells via targeting CKS2. *Int. J. Oncol.* 49 1531–1540. 10.3892/ijo.2016.3660 27633373

[B13] Jin H. F. Wang J. F. Song T. T. Zhang J. Wang L. (2020). MiR-200b inhibits tumor growth and chemoresistance via targeting p70S6K1 in lung cancer. *Front. Oncol.* 10:643. 10.3389/fonc.2020.00643 32435616 PMC7218114

[B14] Krutzfeldt J. Poy M. N. Stoffel M. (2006). Strategies to determine the biological function of microRNAs. *Nat. Genet.* 38(Suppl.), S14–S19. 16736018 10.1038/ng1799

[B15] Le A. Cooper C. R. Gouw A. M. Dinavahi R. Maitra A. Deck L. M. (2010). Inhibition of lactate dehydrogenase A induces oxidative stress and inhibits tumor progression. *Proc. Natl. Acad. Sci. U.S.A.* 107 2037–2042. 10.1073/pnas.0914433107 20133848 PMC2836706

[B16] Li W. Wang L. Ji X. B. Wang L. H. Ge X. Liu W. T. (2019). MiR-199a inhibits tumor growth and attenuates chemoresistance by targeting K-RAS via AKT and ERK signalings. *Front. Oncol.* 9:1071. 10.3389/fonc.2020.01071 31681604 PMC6803549

[B17] Ling H. Fabbri M. Calin G. A. (2013). MicroRNAs and other non-coding RNAs as targets for anticancer drug development. *Nat. Rev. Drug Discov.* 12 847–865. 10.1038/nrd4140 24172333 PMC4548803

[B18] Lu J. Getz G. Miska E. A. Alvarez-Saavedra E. Lamb J. Peck D. (2005). MicroRNA expression profiles classify human cancers. *Nature* 435 834–838. 10.1038/nature03702 15944708

[B19] Luo J. Li H. Zhang C. (2015). MicroRNA-7 inhibits the malignant phenotypes of nonsmall cell lung cancer in vitro by targeting Pax6. *Mol. Med. Rep.* 12 5443–5448. 10.3892/mmr.2015.4032 26135959

[B20] Niu X. B. Fu G. B. Wang L. Ge X. Liu W. T. Wen Y. Y. (2017). Insulin-like growth factor-I induces chemoresistence to docetaxel by inhibiting miR-143 in human prostate cancer. *Oncotarget* 8 107157–107166. 10.18632/oncotarget.22362 29291019 PMC5739804

[B21] Pan H. Li T. Jiang Y. Pan C. Ding Y. Huang Z. (2018). Overexpression of circular RNA ciRS-7 abrogates the tumor suppressive effect of miR-7 on gastric cancer via PTEN/PI3K/AKT signaling pathway. *J. Cell. Biochem.* 119 440–446. 10.1002/jcb.26201 28608528

[B22] Sheng S. L. Liu J. J. Dai Y. H. Sun X. G. Xiong X. P. Huang G. (2012). Knockdown of lactate dehydrogenase A suppresses tumor growth and metastasis of human hepatocellular carcinoma. *FEBS J.* 279 3898–3910. 10.1111/j.1742-4658.2012.08748.x 22897481

[B23] Shi Y. Luo X. Li P. Tan J. Wang X. Xiang T. (2015). miR-7-5p suppresses cell proliferation and induces apoptosis of breast cancer cells mainly by targeting REGgamma. *Cancer Lett.* 358 27–36. 10.1016/j.canlet.2014.12.014 25511742

[B24] Shi Z. Chen Q. Li C. Wang L. Qian X. Jiang C. (2014). MiR-124 governs glioma growth and angiogenesis and enhances chemosensitivity by targeting R-Ras and N-Ras. *Neurol. Oncol.* 16 1341–1353. 10.1093/neuonc/nou084 24861879 PMC4165420

[B25] Vander Heiden M. G. Cantley L. C. Thompson C. B. (2009). Understanding the Warburg effect: the metabolic requirements of cell proliferation. *Science* 324 1029–1033. 10.1126/science.1160809 19460998 PMC2849637

[B26] Wang J. Wang H. Liu A. Fang C. Hao J. Wang Z. (2015). Lactate dehydrogenase A negatively regulated by miRNAs promotes aerobic glycolysis and is increased in colorectal cancer. *Oncotarget* 6 19456–19468. 10.18632/oncotarget.3318 26062441 PMC4637298

[B27] Wang L. Shi Z. M. Jiang C. F. Liu X. Chen Q. D. Qian X. (2014). MiR-143 acts as a tumor suppressor by targeting N-RAS and enhances temozolomide-induced apoptosis in glioma. *Oncotarget* 5 5416–5427. 10.18632/oncotarget.2116 24980823 PMC4170647

[B28] Xiao X. Huang X. Ye F. Chen B. Song C. Wen J. (2016). The miR-34a-LDHA axis regulates glucose metabolism and tumor growth in breast cancer. *Sci. Rep.* 6:21735. 10.1038/srep21735 PMC4763192 26902416

[B29] Xin L. Liu L. Liu C. Zhou L. Q. Zhou Q. Yuan Y. W. (2020). DNA-methylation-mediated silencing of miR-7-5p promotes gastric cancer stem cell invasion via increasing Smo and Hes1. *J. Cell. Physiol.* 235 2643–2654. 10.1002/jcp.29168 31517391

[B30] Yang Y. Su D. Zhao L. Zhang D. Xu J. Wan J. (2014). Different effects of LDH-A inhibition by oxamate in non-small cell lung cancer cells. *Oncotarget* 5 11886–11896. 10.18632/oncotarget.2620 25361010 PMC4323009

[B31] Yang Z. Shi X. Li C. Wang X. Hou K. Li Z. (2018). Long non-coding RNA UCA1 upregulation promotes the migration of hypoxia-resistant gastric cancer cells through the miR-7-5p/EGFR axis. *Exp. Cell Res.* 368 194–201. 10.1016/j.yexcr.2018.04.030 29723509

[B32] Zhao X. Dou W. He L. Liang S. Tie J. Liu C. (2013). MicroRNA-7 functions as an anti-metastatic microRNA in gastric cancer by targeting insulin-like growth factor-1 receptor. *Oncogene* 32 1363–1372. 10.1038/onc.2012.156 22614005

[B33] Zheng T. Wang J. Chen X. Liu L. (2010). Role of microRNA in anticancer drug resistance. *Int. J. Cancer* 126 2–10. 10.1002/ijc.24782 19634138

