# Supplementary material for: Down-Regulation of miR-7 in Gastric Cancer Is Associated With Elevated LDH-A Expression and Chemoresistance to Cisplatin
Source: Front Cell Dev Biol. 2020 Sep 22;8:555937. doi: 10.3389/fcell.2020.555937 (PMC7536350; doi:10.3389/fcell.2020.555937)

Supplementary data

Sup Figure 1. Overexpression of miR-7 suppresses cell proliferation activity. (A, B) Representative photomicrographs and quantifications of EdU immunofluorescence staining assay in BGC823 and SGC7901 cells. Data were presented by mean±SD. of 3 replicates. ** indicated $P<0.01$.

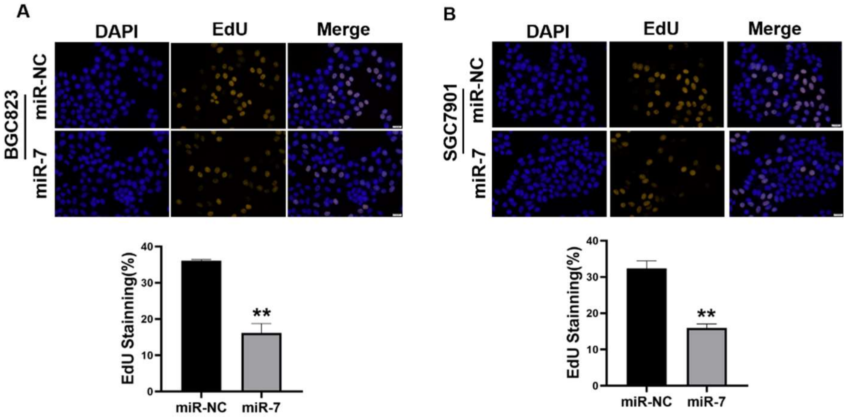

Sup Figure 2. Inhibition of miR-7 in BGC823 induced cell proliferation, colony formation activity and lactate production. (A) Inhibition of miR-7 increased cell growth activity. **(B)** The activity of colony formation were induced in miR-7-inhibitor group. **(C)** Inhibition of miR-7 promoted lactate production. Data were presented by mean±SD. of 3 replicates. * indicated $P<0.05$. ** indicated $P<0.01$.

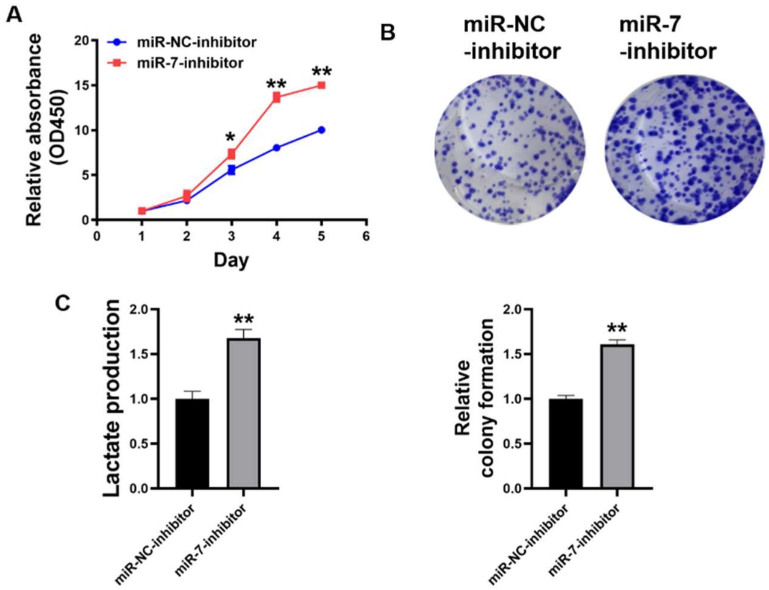

Sup Figure 3. Knockdown with LDHA siRNA decreased cell proliferation, colony formation activity, and lactate production activity. (A) Knockdown with LDHA siRNA decreased cell growth activity in BGC823 cell. **(B)** Colony formation assay were conducted in indicated cells, and the activity of colony formation were reduced in LDHA Knockdown group. **(C)** Inhibition of LDHA reduced lactate production. Data were presented by mean±SD. of 3 replicates. * indicated $P<0.05$. ** indicated $P<0.01$.

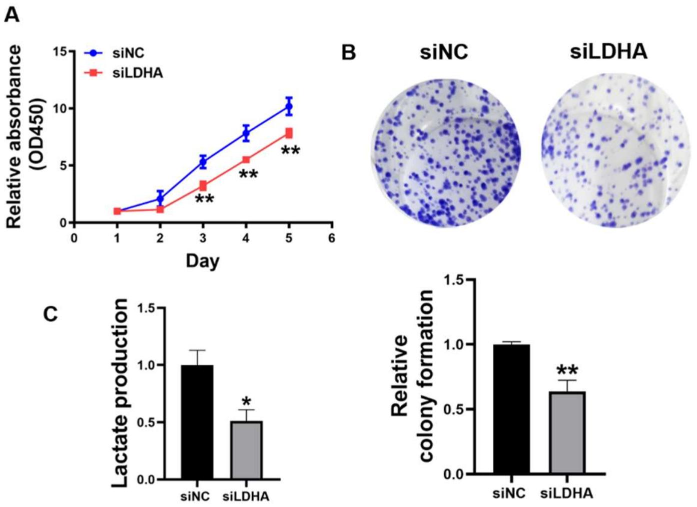

Supplement: Supplementary file 1 [file Image_1.pdf]
